# Morphological and Skeletal Abnormalities Induced by Rolapitant: An Antiemetic Agent

**DOI:** 10.7759/cureus.28097

**Published:** 2022-08-17

**Authors:** Sohel Solanki, Yogesh Yadav, Shaktibala Dutta, Nisha V Kaul, Rashmi Belodu, Hareesh RS Kumar

**Affiliations:** 1 Department of Anatomy, Santosh Medical College, Santosh University, Delhi NCR, IND; 2 Department of Pharmacology, Santosh Medical College, Santosh University, Delhi NCR, IND; 3 Department of Microbiology, Dr. N. Y. (Nandkumar Yadavrao) Tasgaonkar Institute of Medical Science, Raigad, IND; 4 Department of Forensic Medicine and Toxicology, Government Medical College and Associated Hospital, Barmer, IND

**Keywords:** weight and crown-rump length, teratogenic effect, antiemetic agent, skeletal abnormalities, morphological abnormalities, chick embryos, rolapitant

## Abstract

Introduction

Rolapitant hydrochloride is a highly selective, long-acting antagonist of the neurokinin-1 (NK1RA) receptor with a high level of central nervous system (CNS) penetrance. Clinically, it is given to cancer patients with high and moderate emetogenic chemotherapy to prevent chemotherapy-induced nausea and vomiting (CINV). The facts produced in this research support the interpretation of teratogenic effects like anatomical malformations and abnormal skeletal changes affected by high doses of rolapitant in developed chick embryos, which can be extrapolated to humans due to gaps in the literature regarding the teratogenic potential of rolapitant. As rolapitant is metabolized by the liver and excreted through the kidney by leaving a deep impact on the various systems of the body and due to its high plasma concentration with a half-life of more than 180 hours, the study was conducted to acquire some additional information about its adverse effects over the various body systems. Chick is one of the best animals for embryological laboratory research. For various reasons, it is preferred to research embryology in chicks or domestic hens (*Gallus domesticus*). Chick eggs are large, readily available all year, easy to incubate, and regulate artificially.

Aim

This study aimed to determine the morphological and skeletal abnormalities due to the effect of rolapitant, an antiemetic agent, in developing White Leghorn (*G. domesticus*) chicken eggs.

Materials and methods

The experiment used 300 fertilized White Leghorn chicken eggs. The eggs were categorized into five experimental groups (A, B, C, D, and E, each with 30 eggs) and five control groups (a, b, c, d, and e, each with 30 eggs). Rolapitant was administered into the five experimental groups of eggs on incubation day five at various concentrations of 0.00039, 0.0005, 0.00075, 0.001, and 0.00125 mg, respectively, while the control groups received the same volume (0.039, 0.05, 0.075, 0.1, and 0.125 ml, respectively) of normal saline.

Results

At all doses, the mean weight and crown-rump (CR) length of chick embryos were significantly greater in the control group than in the experimental group. The experimental group died at a higher rate than the control group. Additionally, it was found that the mortality due to the rolapitant dosages increases with dose. All groups except group A showed skeletal anomalies such as poor ossification, bent, and displacement, and morphological abnormalities such as yolk sac retraction, hematoma, and scanty feathers were found in experimental groups C, D, and E. This was shown to be more prevalent in the experimental groups and was exacerbated by subsequent rolapitant dosages.

Conclusion

Rolapitant is toxic when taken in large doses and for an extended period. As a result, rolapitant should be taken only when a valid diagnosis has been established and only at the recommended dose, not at a larger dose or for an extended period of time.

## Introduction

Rolapitant's pharmacological capabilities as a highly competitive neurokinin-1 (NK-1) inhibitor were established in animal models through pharmacokinetic investigations. The lack of significant drug-drug interactions is rolapitant's main pharmacokinetic benefit over other NK-1 receptor antagonists. Rolapitant was supplemented to 5-hydroxytryptamine receptor antagonist (5-HT3RA) dexamethasone combination in patients undergoing moderately emetogenic cancer chemotherapy (MEC) or extremely emetogenic cancer chemotherapy/high emetogenic cancer chemotherapy (HEC). Rolapitant has also been shown to protect against chemotherapy-induced nausea and vomiting (CINV) throughout the high-risk phase after chemotherapy, corresponding to the drug's pharmacokinetics [1].

Rolapitant hydrochloride is a long-acting, extremely selective antagonist of the Nk-1 receptor antagonist (NK-1RA) that significantly penetrates the central nervous system (CNS). In 2015, the Food and Drug Administration (FDA) authorized rolapitant, a new NK1RA inhibitor, to treat delayed CINV. Its half-life is 180 hours, which is much longer than that of oral aprepitant (9-13 hours). Rolapitant has been licensed to treat delayed nausea and vomiting induced by emetogenic cancer chemotherapy but is not limited to extremely emetogenic chemotherapy when combined with additional antiemetic medications [2].

Rolapitant is rapidly absorbed and detectable in plasma after 30 minutes of oral administration; the maximum plasma concentration (Cmax) is obtained after four hours. The increases in dose-enhanced rolapitant's systemic exposure (a four-fold increase from the recommended therapeutic dose of 180 mg) increased the Cmax and area under the curve of rolapitant by 3.1 and 3.7 times, respectively. However, fatty meal intake did not influence its absorption [3].

While cytochrome P450 (CYP) enzyme activation is the cause of the majority of observed drug-drug interactions through another NK-1RA, the degree of interaction varies, necessitating dose changes for concomitant drugs like dexamethasone [4]. The cytochrome P450 3A4 (CYP3A4) enzyme is metabolized by rolapitant, although it does not inhibit or activate it. Rolapitant should not be taken with rifampicin, a CYP3A4 inducer; however, it does have a minor inhibitory effect on cytochrome P450 2D6 (CYP2D6), breast cancer resistance protein (BCRP), and P-glycoprotein (P-gp) [5].

The facts produced in this research support the interpretation of teratogenic effects like anatomical malformations and abnormal skeletal changes affected by high doses of rolapitant in developed chick embryos, which can be extrapolated to humans due to gaps in the literature regarding rolapitant's teratogenic potential. As it is metabolized by the liver and excreted through the kidneys by leaving a deep impact on the various systems of the body and due to its high plasma concentration with a half-life of more than 180 hours, the study was conducted to acquire some additional information about its adverse effects over the various body systems. This study aimed to see if rolapitant, an antiemetic medicine, may cause teratogenic effects in developing White Leghorn (*Gallus domesticus*) chicken eggs. To gain a comprehensive understanding of embryological processes, researchers must examine and compare development in various animals. The chick is one of the best animals for embryological laboratory research. For various reasons, it is preferred to research embryology in chicks or domestic hens (*G. domesticus*). Chick eggs are large, readily available all year, easy to incubate, and regulate artificially. Furthermore, the process of chicken growth has been well investigated. An examination of the embryology of various birds reveals that it is almost identical in all of them, with just minor variations. Chicken embryology is quite similar to human embryology. Without a larval stage, the development proceeds directly [6].

## Materials and methods

Study area

This study aims to see whether rolapitant's antiemetic effect on chick embryo development induced abnormal morphological and skeletal effects on 300 (out of 330 eggs) White Leghorn chicken eggs. The research was accomplished in the Department of Anatomy at Santosh Medical College in Ghaziabad, Uttar Pradesh. The eggs were provided by Metro Feeds, Indotech Agro Products, Faridabad, India.

Inclusion and exclusion criteria

The well-calcified eggs were included with an entire shell, an air cell with a broad end, and an egg's air cell free of blood clots. Thirty eggs were rejected due to cracked shells (n = 17), lack of an air cell at the broad end (n = 8), and an air cell with a blood clot (n = 5).

Procedure

Natekar’s [7] standard approach was used to conduct the study. Candles were used to obliterate faulty eggs, and a pencil was used to mark the precise location of the air cell. A custom-built wooden box with a compact fluorescent light (CFL) connector and a dark interior was employed. There are spaces for chicken eggs on the top. After being well cleansed with soap water, the eggs were kept in the incubator with their broad end facing the chorioallantoic membrane. The incubator's temperature and humidity were kept at 38°C and 85%-90%, respectively, without the addition of CO_2_ (carbon dioxide) or O_2_ (oxygen). Twice a day, the eggs were inverted. Eggs were taken from the incubator and lit with candles again on the fifth day of incubation to determine the start of the embryogenesis process before being given the drug. Natekar’s approach [7] was used by Singroha et al. (2012) [8], Thongphanich and Roongruangchai (2013) [9], Hussein (2014) [10], and Mittal et al. (2015) [11].

Dose setting

The recommended dose of rolapitant for adults, as approved by the US FDA, is 180 mg via the oral route, i.e., 3 mg/kg of average body weight (60 kg). On the fifth day of incubation, the weight of a chick embryo was 0.13 grams [12]. Details of dose titration for experimental and control groups of chick embryos are as follows (Table 1).

**Table 1:** Dose preparation for chick embryos for the administration of rolapitant into the five experimental groups N = 30 in each experimental (n1) and control groups (n2). NS: Normal saline; mg: milligram; ml: milliliter; Unit: measured by tuberculin syringe.

Groups	Experimental groups		Control groups	
	Dose (rolapitant)	Sample size (n1)	Dose (NS)	Sample size (n2)
A	0.00039 mg	30	0.039 ml/1.56 Units	30
B	0.0005 mg	30	0.05 ml/2 Units	30
C	0.00075 mg	30	0.075 ml/3 Units	30
D	0.001 mg	30	0.1 ml/4 Units	30
E	0.00125 mg	30	0.125 ml/5 Units	30

Dose titration

Calculated dose of drug for chick embryo = 3 × .13/1000 = 0.00039 mg (on incubation day 5). The following method was adopted to prepare the doses. Rolapitant power contains 10 mg of drug dissolved in 10 ml of distilled water (called solution A). Step 1: 1 ml of solution A was taken in a test tube; 1 ml of solution A = 1 mg of the drug. Step 2: 1 ml of solution A diluted with 9 ml distilled water to get 10 ml of solution B. 1 ml of solution B now contains 0.1 mg of the drug. Step 3: 1 ml of solution B diluted with 9 ml of distilled water to get 10 ml of solution C. 1 ml of solution C now contains 0.01 mg of the drug. *0.039 ml of solution C contain 0.00039 mg of the drug (0.039 ml × 0.01 mg). Dose titration: To convert ml into unit of insulin syringe. *1 ml insulin syringe contains 40 units. *1 unit insulin syringe contains 0.025ml (1/40). *0.039 ml (0.00039 mg) contains (0.039/0.025 ml) 1.56 units. The following seven units have different doses of drugs: 1 unit contains 0.00039 mg/1.56 unit = 0.00025 mg of the drug; 1.5 units contain 0.00039 mg of the drug; 2 units contain (0.00039 × 2/1.56) = 0.0005 mg of the drug; 3 units contain (0.00039 x 3/1.56) = 0.00075 mg of the drug; 4 units contain (0.00039 × 4/1.56) = 0.001 mg of the drug; 5 units contain (0.00039 × 5/1.56) = 0.00125 mg of the drug; 6 units contain (0.00039 × 6/1.56) = 0.0015 mg of the drug (lethal dose 50%). More than 50% of embryos were dead at this dose.

*A preliminary drug study was done to obtain a lethal dose of 50% (LD50). The dose of 0.0015 mg was found as LD50, at which more than 50% of chick embryos were found dead. *All concentrations of drug below LD50 was selected for the study, namely, 0.00039, 0.0005, 0.00075, 0.001, and 0.00125 mg except the dose of 0.00025 mg, at which no apparent abnormalities were detected. This is why we had chosen the above-selected doses in the present study.

Method for drug administration, collection, and preservation of chick embryos

**Figure 1:**
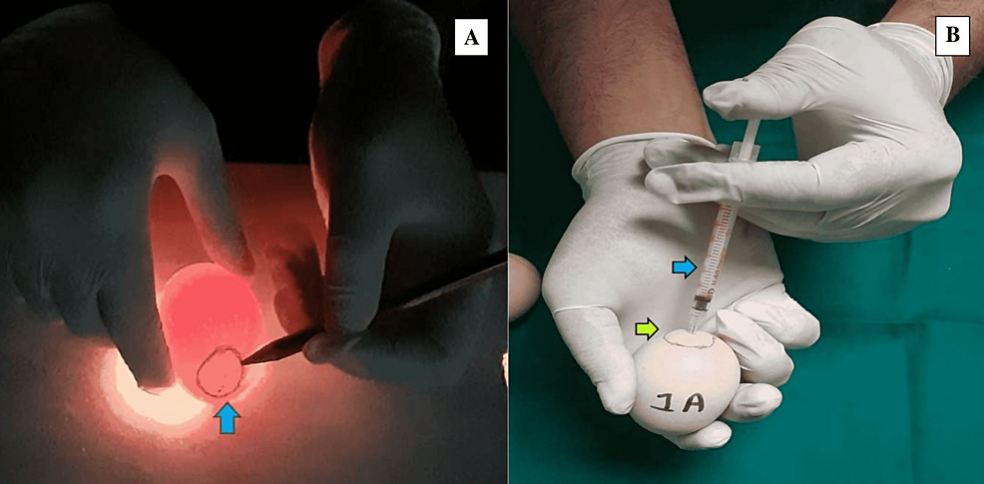
Method of drug administration (A) Peripheral marking of the exact location of the air shell with pencil on the outer broader surface of an egg (blue arrow). (B) Drug administration by introducing the middle of tuberculin syringe (blue arrow) into the air shell (green arrow).

The eggs were categorized into five experimental groups, designated as A, B, C, D, and E. Each group contained 30 eggs, and five control groups, a, b, c, d, and e, each containing 30 eggs. As seen in Table 1, various doses were administered. The eggs were shackled three to four times between two hands. The fluid was administered into the air sac with the help of an insulin syringe. The wide end of the eggs was cleansed with a sterile gauze pad saturated in 70% isopropyl alcohol solution before being placed in the incubator. The outline of the air shell was drawn with a pencil at the peripheral margin, set by keeping eggs against the light coming from a hole made in a specially formed wooden box supplied with an illuminator inside. With a lancet, a hole was punched through the center of the outer surface of the egg's air shell. The drill point was utilized with caution because of the possibility of injuring the shell membranes inside. The drill point was used to insert the tuberculin syringe needle into the shell in a parallel fashion. During the intervals between administrations, the needle of the syringe was wiped with a sterile 70% isopropyl alcohol wipe (Figure 1).

Following injection, the opening in the shell was immediately sealed with candle-melted wax. Eggs that had been sealed after injection were reintroduced to the incubator at 38°C and 85%-90% humidity, where they were tilted twice a day at 38°C and 85%-90% humidity. After that, the eggs were alternately candled with a candle. A rotation of the eggs occurred twice a day. On the 20th day of incubation, the eggs were cracked using a knife to extract embryos for analysis. The number of alive and dead embryos was determined. An electronic weighing machine was used to assess the weight of chick embryos, and digital Vernier calipers were used to measure their crown-rump (CR) length (a distance between the tip of the beak and the end of the coccyx bone). Identification of morphological aberrations was performed using a necked eye and magnifying lens as well as Vernier calipers. Before the start of the investigation, 15 fertilized White Leghorn chicken eggs were established in an incubator without using normal saline or distilled water, or any medicine. On the 20th day of incubation, the eggs were lost; however, chick embryos were retrieved. Their weight and CR length were determined using electronic scales and digital Vernier calipers, respectively. Without providing any solution to the eggs, we determined the normal minimum weight of the embryos to be 32.28 g, the maximum weight to be 39.42 g, the minimum CR length to be 9.93 cm, and the maximum CR length to be 11.35 cm. Chick embryos' mean normal weight and CR length were 35.75 ± 2.15 g and 10.57 ± 0.45 cm, respectively. We used these data to compare the outcomes of the results between the control and experimental groups.

Dissection of chick embryos and staining of the skeleton

To identify skeletal defects in chick embryos, the ventral wall of the trunk was dissected, and all internal organs were removed. Chick embryos that have been eviscerated are preserved in a 10% formaldehyde solution. The eviscerated chick embryos were cleaned with tap water and processed according to McLeod's protocol for double staining cartilage and bone with slight modifications. The embryos were fixed in 100% ethyl alcohol for seven days. Following fixation, embryos were stained for four days in a solution containing (a) 1 volume 0.1% Alizarin Red S in absolute ethyl alcohol, (b) 1 volume glacial acetic acid, and (c) 1 volume absolute ethyl alcohol. A 100-ml solution was used in a separate jar for each skeleton to avoid body parts overlapping to guarantee enough staining and consistency. Following staining, the embryos were rinsed with tap water and incubated for 48 hours in a 2% potassium hydroxide solution. To prevent mold proliferation, macerated pigmented embryos were passed through various percentages of glycerine (20%, 40%, 60%, and 80%) and then kept in pure glycerin containing some thymol crystals. For visualization reasons, photographs were taken in the department's photographic laboratory.

Statistical analysis

Statistical Package for the Social Sciences (SPSS) software v15.0 (SPSS Inc., Chicago, IL) was used for statistical analysis. For dichotomous variables, proportions were employed, whereas for continuous variables, mean and standard deviation were used. Where applicable, the chi-squared test was used to find the proportional connection. The chi-squared trend test was also used to see if there was a link between rising rolapitant doses and other developmental problems. If necessary, continuous variables were compared using the paired/unpaired t-test. A p-value of less than 0.05 was defined as statistically significant.

## Results

**Figure 2:**
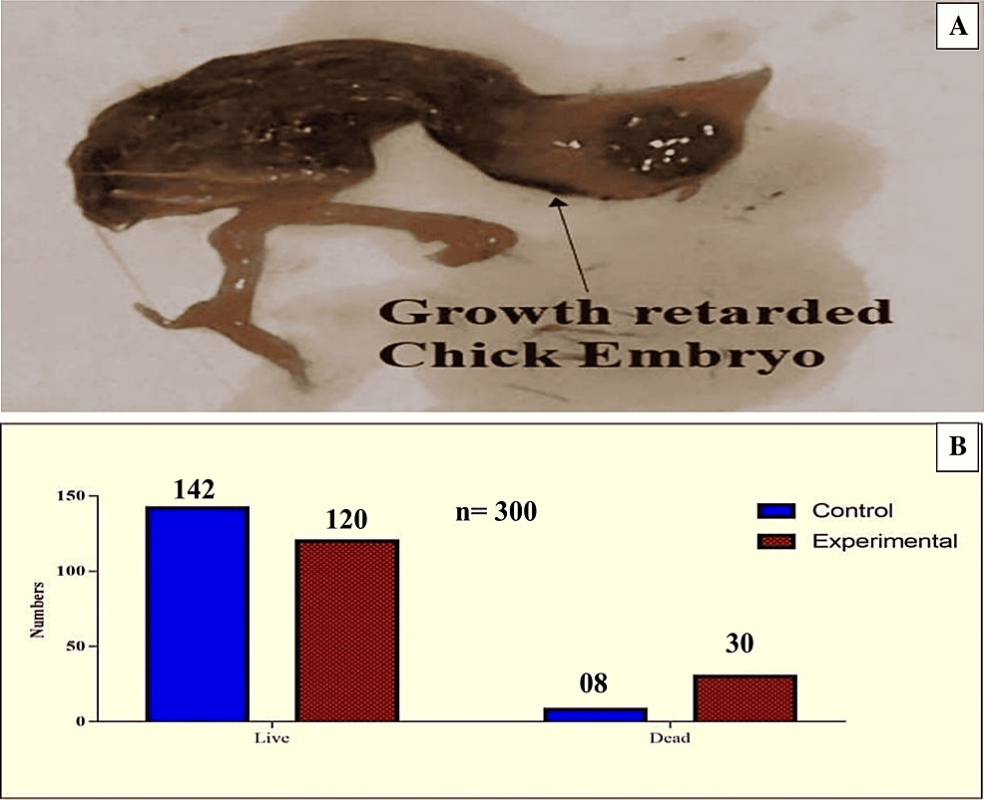
Lethal impact of rolapitant on developing chick embryos (A) Growth-retarded dead chick embryo. (B) Number of alive and dead chick embryos in experimental and control groups induced by rolapitant.

In all doses, the control groups had a higher average weight than the experimental groups and were statistically significant in groups D and E (p-value < 0.05). Similarly, short CR length (CRL) was noted more in a number of chick embryos of experimental groups as compared to control groups and was found statistically significant in groups D and E (p-value < 0.05). The mean weight in the control groups was substantially higher than the mean weight in the experimental groups, except for groups A and B. Similarly, the mean CRL in the control groups was substantially longer than the mean CRL in the experimental groups, except for groups A and B (P-value < 0.05). There were more deaths in the experimental groups than in the controls. The deadly effect of rolapitant demonstrated a subsequent considerable rise in lethality with the dose increase. While analyzing the lethal impact of the drug on a number of chick embryos, the majority of the death occurred in group D (p = 0.0377) and E (p = 0.0073), which showed a statistically significant difference. The total number of dead chick embryos in the control and experimental groups was noted as eight and 30, respectively (Figure 2 and Tables 2-4).

**Table 2:** Number of chick embryos with weight and CRL due to the effects of rolapitant N: 30 eggs in each group; a, b, c, d, and e: control groups; A, B, C, D, and E: experimental groups; CRL: crown-rump length.

Name of the group	Number of chicken born with normal weight	Number of chicken born with less than normal weight	Number of chicken born with more than normal weight	Number of chicken born with normal CRL	Number of chicken born with less than normal CRL	Number of chicken born with more than normal CRL
a	28	1	1	28	1	1
A	26	3	1	27	2	1
b	28	1	1	27	2	1
B	26	3	1	25	4	1
c	26	2	2	26	3	1
C	23	6	1	20	8	2
d	26	3	1	24	5	1
D	20	10	0	18	12	0
e	27	3	0	25	4	1
E	15	15	0	14	16	0

**Table 3:** Mean, SD, t, and p-values of weight and crown-rump length in experimental and control groups A, B, C, D, and E: Compared groups; t: t-test; p: significant value; SD: standard deviation. * indicates highly significant values.

Groups	Weight		P-value	CR Length		P-value
	Control Mean ± SD	Experiment Mean ± SD		Control Mean ± SD	Experiment Mean ± SD	
A	35.26 ± 2.32	34.92 ± 2.639	t = 0.53, p = 0.5982	10.48 ± 0.57	10.41 ± 0.42	t = 0.4762, p = 0.6357
B	34.93 ± 2.066	33.78 ± 2.257	t = 2.059, p = 0.0439*	10.44 ± 0.43	10.24 ± 0.40	t = 1.841, p = 0.0707
C	34.26 ± 1.948	32.8 ± 2.772	t = 2.365, p = 0.0214*	10.36 ± 0.41	10.02 ± 0.50	t = 2.779, p = 0.0073*
D	33.5045 ± 2.098	31.98 ± 2.168	t = 2.849, p = 0.0061*	10.34 ± 0.44	9.98 ± 0.59	t = 2.624, p = 0.0111*
E	34.58 ± 1.849	31.93 ± 2.728	t = 4.335, p < 0.0001*	10.41 ± 0.50	9.94 ± 0.42	t = 3.887, p = 0.0003*

**Table 4:** Lethal impact induced by rolapitant on developing chick embryo Number of dead and alive chick embryos in both experimental (A, B, C, D, and E) and control groups (a, b, c, d, and e) with chi-square and p-values.

Name of the groups with doses	Number of fertile eggs used	Number of dead embryos	Number of live embryos	Percentile lethality (%)	Chi-square	P-value
a	30	0	30	0	2.069	0.1503
A	30	2	28	6.6		
b	30	1	29	3.3	1.071	0.3006
B	30	3	27	10		
c	30	2	28	6.6	1.456	0.2276
C	30	5	25	16.6		
d	30	2	28	6.66	4.32	0.0377*
D	30	8	22	26.6		
e	30	3	27	10	7.2	0.0073*
E	30	12	18	40		

**Figure 3:**
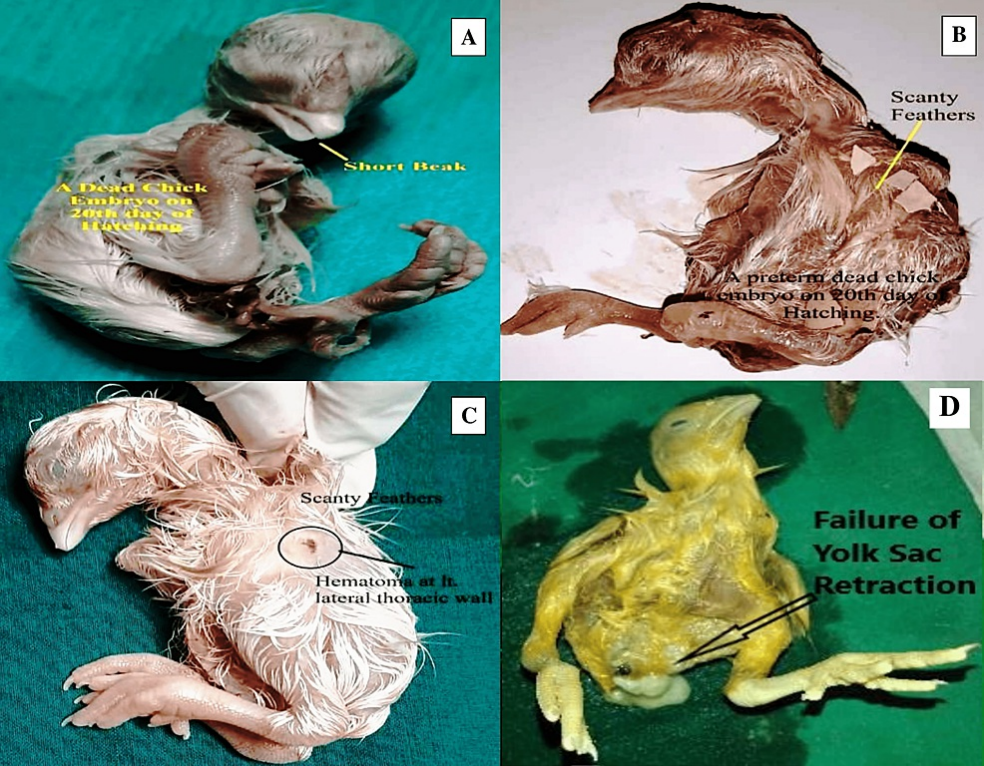
Morphological abnormalities in developing chick embryos induced by rolapitant (A) Dead chick embryo with short beak (yellow arrow), (B) scanty feathers (yellow arrow), (C) hematoma (black circle) on the left lateral thoracic wall of chick embryo with scanty feathers, and (D) failure of retraction of the yolk sac (arrow).

**Figure 4:**
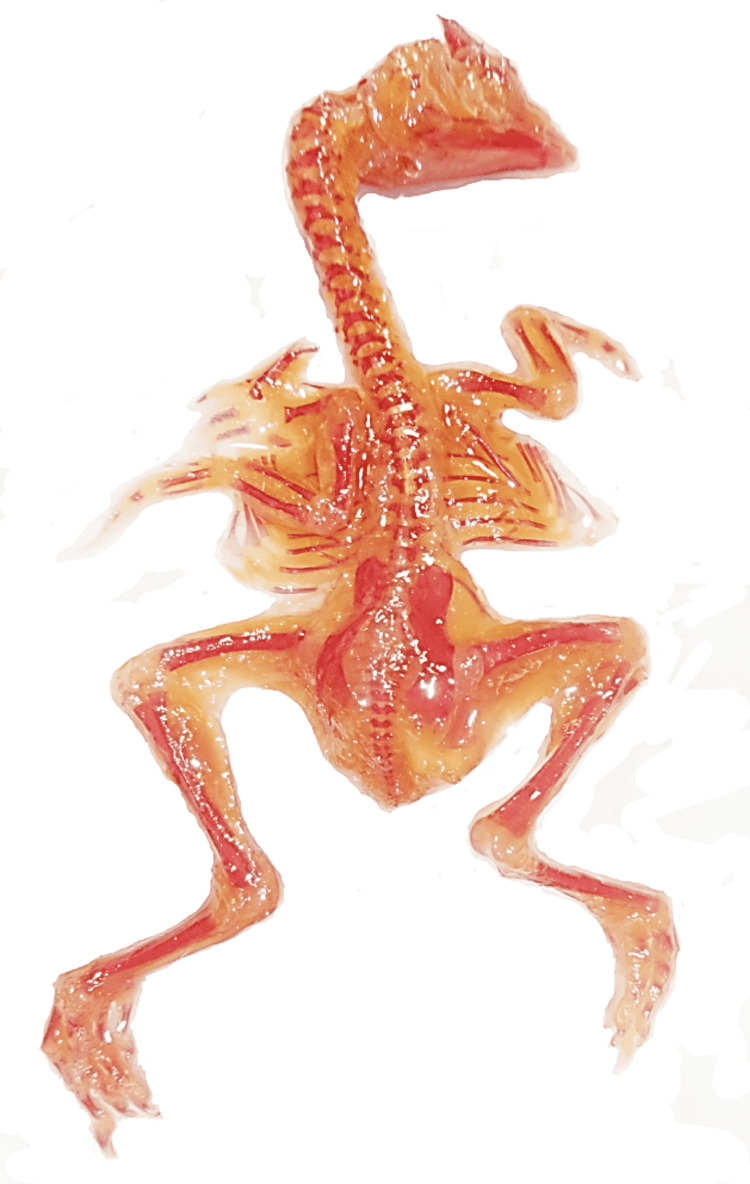
Skeleton of a normal chick embryo: dorsal aspect

**Figure 5:**
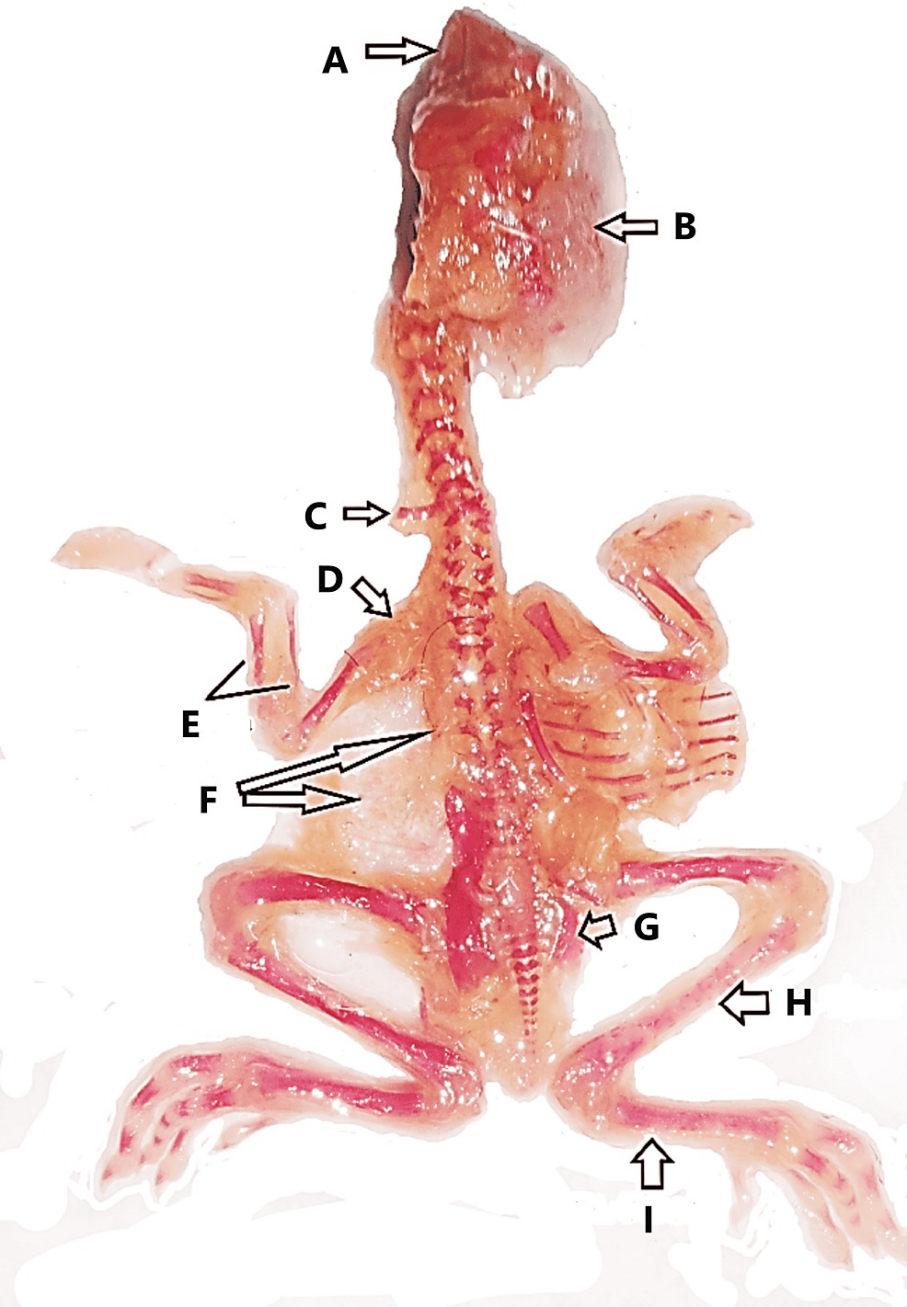
Abnormal skeleton of a chick embryo (A) Poor ossification of mandible and maxilla, (B) poor ossification of skull, (C) cervical rib, (D) poor ossification of left clavicle, (E) poor ossification of radius and ulna with thinning, (F) absence of scapula and ribs (left sided), (G) poor ossification and thinning of right pelvis, (H) poor ossification of right tibia, and (I) poor ossification of right fibula.

**Figure 6:**
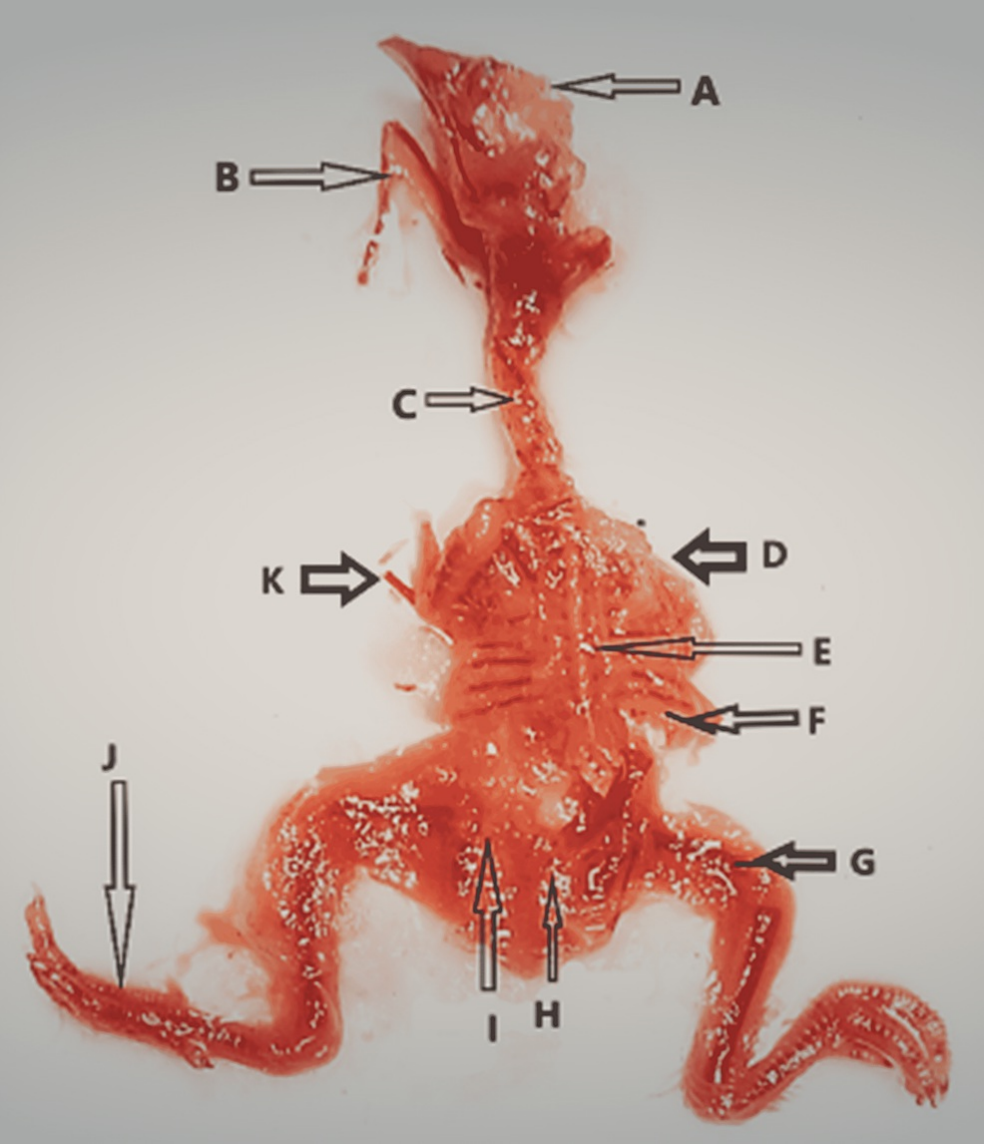
Abnormal skeleton of a chick embryo (A) Poor ossification of skull, (B) short mandible with poor ossification and thinning, (C) poor ossification of cervical vertebrae, (D) absence of right upper limb bones, (E) poor ossification of thoracic vertebrae, (F) poor ossification of ribs and scapula, (G) poor ossification of right femur (short), (H) absence of tail, (I) poor ossification of left pelvis, (J) short fibula wit-fused metatarsals and phalanges, and (K) poor ossification left humerus and absence of left radius, ulna, metacarpals, and phalanges.

**Figure 7:**
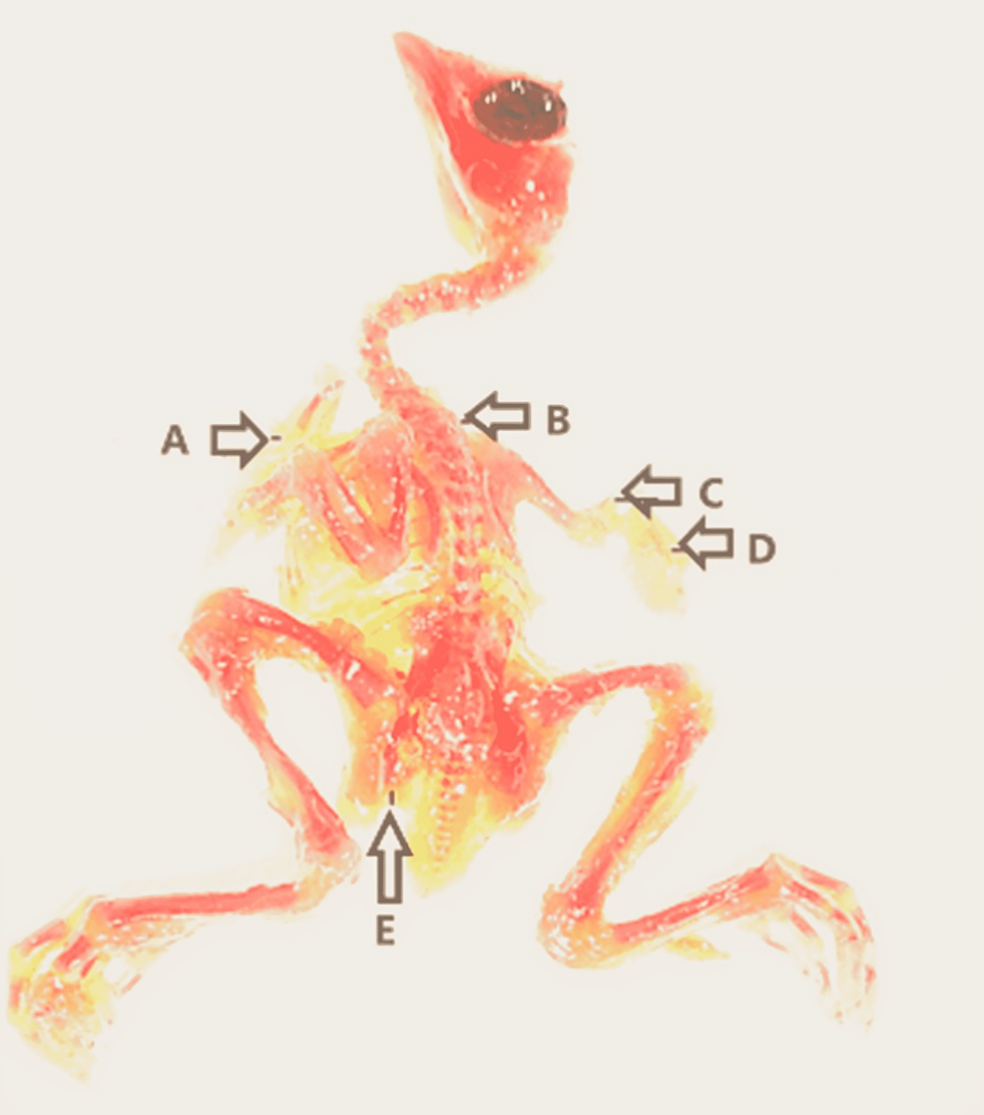
Abnormal skeleton of a chick embryo (A) Undeveloped sternum, (B) scoliosis, (C) poor ossification of right radius and ulna, (D) poor ossification of right metacarpals and phalanges, and (E) poor ossification of left pelvis.

**Figure 8:**
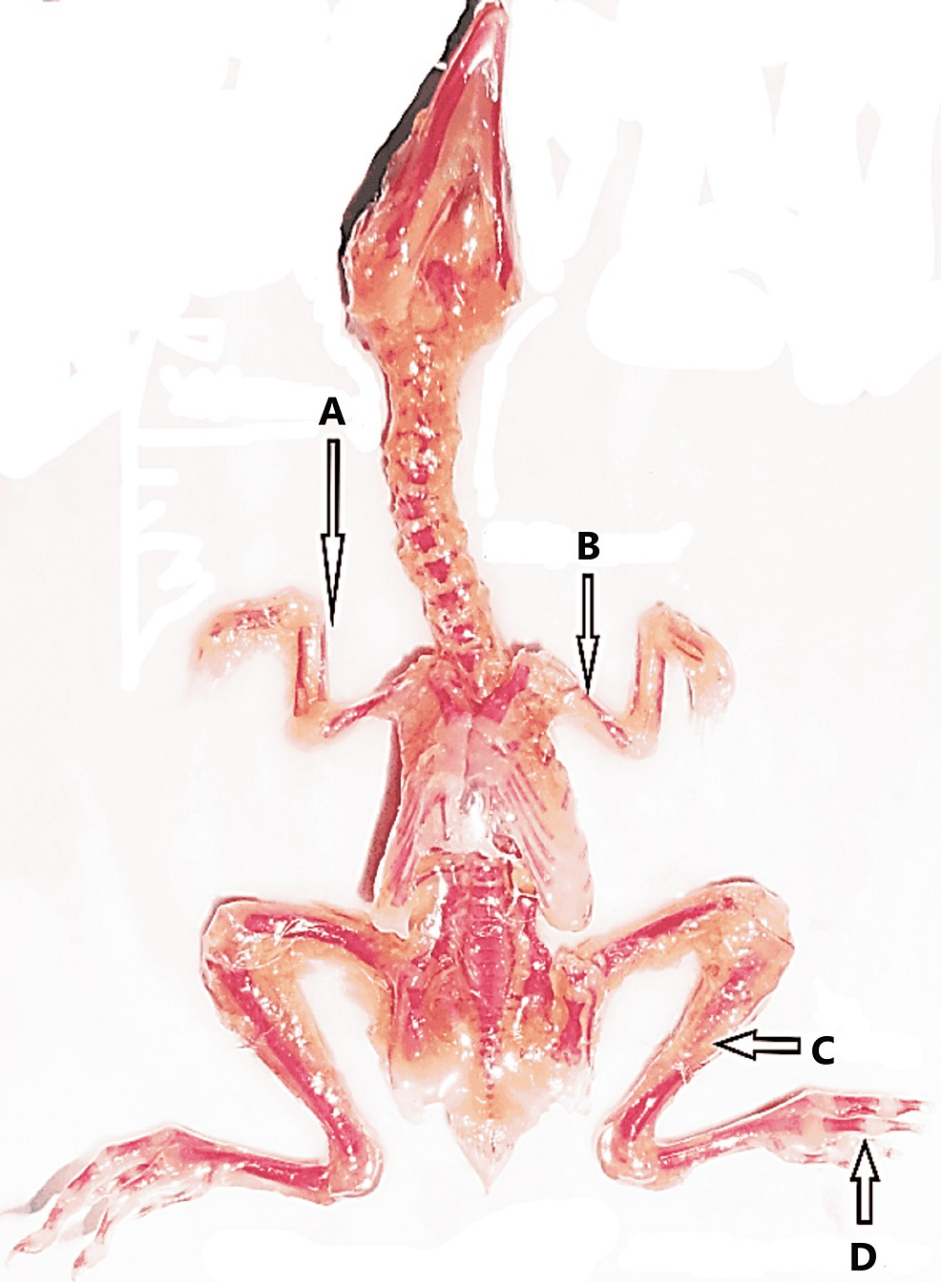
Abnormal skeleton of a chick embryo (A) Poor ossification of right radius and thinning of right ulna, (B) poor ossification of left humerus and thinning of left radius and ulna, (C) poor ossification of left fibula, and (D) poor ossification of phalanges of left toe.

No morphological abnormalities were seen in either the control or experimental groups A and B. The small beak, scanty feathers, hematoma, and yolk sac retraction failure were detected in experimental groups C, D, and E. Prevalence was directly proportionate to a higher dose of the drug. However, most of the morphological abnormalities were determined and found to be significant only in the experimental group E. When the skeleton abnormalities were analyzed, a significant rise in the number of various chick embryos of experimental groups induced by rolapitant was detected. Most of the skeletal anomalies such as poor ossification, bent or displacement, disoriented, thinning, fusion, undeveloped, or absence of the bones of axial and appendicular skeletons were observed after staining of the skeletal. The Alizarin Red with other substitute chemicals was used to stain the bones. Full absorption of staining by bones was noted as complete and normal ossification, those by which staining was absorbed less or partly were demonstrated as poor ossification, and those by which staining was not absorbed were declared as absent (Table 5 and Figures 3-8).

**Table 5:** Number of chick embryos with morphological malformation at different doses in experimental and control groups N = 30 in each group; a, b, c, d, and e: control groups; A, B, C, D, and E: experiment groups.

Groups	Morphological malformations												
	Macrocephaly	Microcephaly	Exophthalmia	Microphthalmia	Narrow neck	Twisted neck	Long beak	Short beak	Hematoma	Limbus deformity	Ectopic viscerum	Scanty feathers	Failure to yolk sac retraction
a	0	0	0	0	0	0	0	0	0	0	0	0	0
A	0	0	0	0	0	0	0	0	0	0	0	0	0
b	0	0	0	0	0	0	0	0	0	0	0	0	0
B	0	0	0	0	0	0	0	0	0	0	0	0	0
c	0	0	0	0	0	0	0	0	0	0	0	0	0
C	0	0	0	0	0	0	0	0	1	0	0	1	1
d	0	0	0	0	0	0	0	0	1	0	0	2	0
D	0	0	0	0	0	0	0	0	4	0	0	7	4
e	0	0	0	0	0	0	0	0	1	0	0	2	1
E	0	0	0	0	0	0	0	1	9	0	0	8	7

In addition to the observation of skeletal anomalies as demonstrated in Figures 5-8, retarded or poor ossification of skull bones was seen in all groups except control groups "a” and “b." Significant changes were observed in the experimental group E only and showed the retarded ossification in 30% and short mandible in 2%. However, the experimental groups showed more skeletal abnormalities in skull bones than the control groups. Skeletal abnormalities in the spinal column, with the exception of group A, and all others exhibited skeletal anomalies such as inadequate ossification or fusion, scoliosis, lordosis, kyphosis, and kinky or absent tail, while cervical rib was detected in group E only. However, it was more prevalent in the experimental group and directly proportionate to high doses of the drug. Rolapitant-induced abnormalities in the ribs such as poor ossification, absence, and synostosis were noted. The anomalies in the sternum such as poor ossification, displaced, and dumbbell shaped were detected. In the upper limb skeleton of chick embryos of the control and experiment groups, no abnormal changes were found in the control groups. While the experimental groups showed significant abnormalities like poor ossification of phalanges, metacarpals, radius, ulna, and carpal bones with thinning of radius, ulna, carpals, and metacarpals, abnormal alterations in the lower limbs such as inadequate ossification, shortening, bending or displacement, and fusion of the foot bones were noted (Tables 6-11).

**Table 6:** Skeletal abnormalities in the skull due to the effects of rolapitant N = 30 in each group. a, b, c, d, and e: control groups; A, B, C, D, and E: experiment groups.

Groups	Retarded ossification	Short maxilla	Short mandible	Congenital absence of cranium	Any other abnormality
a	Nil	Nil	Nil	Nil	Nil
A	1 (3.3%)	Nil	Nil	Nil	Nil
b	Nil	Nil	Nil	Nil	Nil
B	1 (3.3%)	Nil	Nil	Nil	Nil
c	2 (6.66%)	Nil	Nil	Nil	Nil
C	3 (10%)	Nil	Nil	Nil	Nil
d	1 (3.3%)	Nil	Nil	Nil	Nil
D	6 (20%)	Nil	Nil	Nil	Nil
e	1 (3.3%)	Nil	Nil	Nil	Nil
E	9 (30%)	Nil	2 (6.66%)	Nil	Nil

**Table 7:** Skeletal abnormalities in the vertebral column due to the effects of rolapitant N = 30 in each group. a, b, c, d, and e: control groups; A, B, C, D, and E: experiment groups.

Groups	Not/poorly ossified (%)	Absent	Displaced or fused (%)	Scoliosis (%)	Lordosis (%)	Kyphosis (%)	Absent or kinky tail	Any other abnormality
a	Nil	Nil	Nil	Nil	Nil	Nil	Nil	Nil
A	1 (3.3%)	Nil	Nil	Nil	Nil	Nil	Nil	Nil
b	Nil	Nil	Nil	Nil	Nil	Nil	Nil	Nil
B	1 (3.3%)	Nil	Nil	1 (3.3%)	Nil	Nil	Nil	Nil
c	1 (3.3%)	Nil	Nil	Nil	Nil	Nil	Nil	Nil
C	4 (13.33%)	Nil	Nil	Nil	1 (3.3%)	1 (3.3%)	Nil	Nil
d	3 (10%)	Nil	Nil	Nil	Nil	Nil	Nil	Nil
D	7 (23.33%)	Nil	Nil	1 (3.3%)	Nil	1 (3.3%)	Nil	Nil
e	2 (6.66%)	Nil	Nil	1 (3.3%)	Nil	1 (3.3%)	Nil	Nil
E	8 (26.66%)	Nil	1 (3.3%)	3 (10%)	Nil	2 (6.66%)	1 (3.3%)	Nil

**Table 8:** Abnormalities in the rib due to the effects of rolapitant N = 30 in each group. a, b, c, d, and e: Control groups; A, B, C, D, and E: experiment groups.

Groups	Incomplete or poor ossification	Absent or displaced	Synostosis	Any other abnormality
a	Nil	Nil	Nil	Nil
A	2 (6.66%)	1 (3.33%)	Nil	Nil
b	Nil	Nil	Nil	Nil
B	3 (10%)	1 (3.33%)	Nil	Nil
c	2 (6.66%)	Nil	Nil	Nil
C	5 (16.66%)	1 (3.33%)	Nil	Nil
d	1 (3.33%)	1 (3.33%)	Nil	Nil
D	8 (26.66%)	2 (6.66%)	Nil	Nil
e	2 (6.66%)	1 (3.33%)	Nil	Nil
E	12 (40%)	3 (10%)	1 (3.33%)	Nil

**Table 9:** Various abnormalities in the sternum in experimental and control groups with the number of chick embryos N = 30 in each group. a, b, c, d, and e: Control groups; A, B, C, D, and E: experiment groups.

Groups	Poorly or not ossified (%)	Displaced (%)	Dumbbell shaped (%)	Synostosis (%)	Asternia (%)	Any other abnormality
a	Nil	Nil	Nil	Nil	Nil	Nil
A	1 (3.33%)	Nil	Nil	Nil	Nil	Nil
b	Nil	Nil	Nil	Nil	Nil	Nil
B	1 (3.33%)	1 (3.33%)	Nil	Nil	Nil	Nil
c	2 (6.66%)	Nil	Nil	Nil	Nil	Nil
C	5 (16.66%)	1 (3.33%)	Nil	Nil	Nil	Nil
d	1 (3.33%)	Nil	Nil	Nil	Nil	Nil
D	7 (23.33%)	1 (3.33%)	1 (3.33%)	Nil	Nil	Nil
e	1 (3.33%)	Nil	Nil	Nil	Nil	Nil
E	11 (36.66%)	2 (6.66%)	2 (6.66%)	Nil	Nil	Nil

**Table 10:** Abnormal changes in the skeleton of upper limb exposed to rolapitant N = 30 in each group. a, b, c, d, and e: Control groups; A, B, C, D, and E: experiment groups.

Name of the bones	Control group																			
	Poorly ossified or not ossified (%)					Shortened					Bent or displacement					Any other change				
	a	b	c	d	e	a	b	c	d	e	a	b	c	d	e	a	b	c	d	e
Clavicle	No	No	No	No	1	No	No	No	No	No	No	No	No	No	No	No	No	No	No	No
Scapula	No	No	No	1	1	No	No	No	No	No	No	No	No	No	No	No	No	No	No	No
Humerus	No	No	1	1	1	No	No	No	No	No	No	No	No	No	1	No	No	No	No	No
Radius	No	No	1	1	2	No	No	No	No	No	No	No	No	No	No	No	No	No	No	No
Ulna	No	No	No	1	2	No	No	No	No	No	No	No	No	No	No	No	No	No	No	No
Carpals	No	No	No	No	1	No	No	No	No	No	No	No	No	No	No	No	No	No	No	No
Metacarpals	No	1	1	1	2	No	No	No	No	1	No	No	No	No	No	No	No	No	No	No
Phalanges	No	1	1	1	2	No	No	No	No	2	No	No	No	No	No	No	No	No	No	No
Experimental group																				
Name of the bones	Poorly ossified or not ossified (%)					Shortened					Bent or displacement					Any other change				
	A	B	C	D	E	A	B	C	D	E	A	B	C	D	E	A	B	C	D	E
Clavicle	No	No	1	3	4	No	No	No	No	No	No	No	No	1	2	No	No	No	No	No
Scapula	No	No	2	5	4	No	No	No	No	No	No	No	No	2	2	No	No	No	No	No
Humerus	No	No	2	4	5	No	No	No	No	No	No	No	No	1	1	No	No	No	No	No
Radius	2	2	3	6	6	No	No	No	No	1	No	1	1	2	2	No	No	No	Thin	No
Ulna	1	1	No	1	6	No	No	No	No	1	No	No	No	No	1	No	No	No	Thin	No
Carpals	1	2	No	No	3	No	No	No	No	No	No	No	2	1	No	No	No	No	No	Thin
Metacarpals	2	2	3	4	5	No	No	1	No	No	No	No	1	1	2	No	No	No	No	Thin
Phalanges	3	2	2	5	5	No	No	1	No	4	No	No	No	1	4	No	No	No	No	Thin

**Table 11:** Abnormal changes in the skeleton of lower limb exposed to rolapitant N = 30 in each group. a, b, c, d, and e: Control groups; A, B, C, D, and E: experiment groups.

Name of the bones	Control group																			
	Poorly ossified or not ossified (%)					Shortened					Bent or displacement					Any other change				
	a	b	c	d	e	a	b	c	d	e	a	b	c	d	e	a	b	c	d	e
Pelvis	No	1	1	1	1	No	No	No	No	No	No	No	No	No	1	No	No	No	No	No
Femur	No	No	1	No	No	No	No	No	No	No	No	No	No	1	1	No	No	No	No	No
Tibia	No	1	No	No	1	No	No	No	No	No	No	No	No	No	No	No	No	No	No	No
Fibula	No	No	No	1	No	No	No	No	No	1	No	No	No	No	No	No	No	No	No	No
Tarsals	No	No	No	No	No	No	No	No	No	No	No	No	No	No	No	No	No	No	No	No
Metatarsals	1	No	1	1	2	No	No	No	1	No	No	No	No	No	No	No	No	No	No	No
Toes	1	No	1	1	2	No	No	No	1	No	No	No	No	No	No	No	No	No	No	No
Experimental group																				
Name of the bones	Poorly ossified or not ossified (%)					Shortened					Bent or displacement					Any other change				
	A	B	C	D	E	A	B	C	D	E	A	B	C	D	E	A	B	C	D	E
Pelvis	2	2	3	5	10	No	2	No	1	3	No	No	1	2	4	No	Thin	Thin	Thin	Thin
Femur	3	1	3	7	8	1	No	No	1	3	1	2	1	4	5	No	Thin	No	Thin	Thin
Tibia	No	2	2	6	8	No	No	No	1	4	No	1	1	2	4	No	No	No	No	No
Fibula	2	1	4	4	9	No	No	No	No	2	No	No	1	No	2	No	No	No	No	Thin
Tarsals	No	No	No	No	No	No	No	No	2	1	No	No	No	No	3	No	No	No	No	No
Metatarsals	2	1	3	4	7	No	No	1	1	No	No	2	No	1	4	No	No	Thin	Fused	No
Toes	2	1	4	4	8	No	No	2	2	3	2	2	2	1	5	No	No	No	Fused	No

A detailed description of the fatal and unfavorable effects of various doses of rolapitant at higher doses was seen as statistically significant in the experimental groups when compared with control groups. Statistically, chick embryos with abnormal weight was found highly significant in group D (p-value = 0.0283) and group E (p-value = 0.0007), with abnormal CRL found as highly significant in group D (p-value = 0.0195) and group E (p-value = 0.0029), with abnormal skull bone found highly significant in only group E (p-value = 0.0056), with abnormal vertebral column found highly significant in only group E (p-value = 0.0377), with abnormal ribs found highly significant in group D (p-value = 0.001) and group E (p-value = 0.002), with abnormal sternum found highly significant in group D (p-value = 0.022) and group E (p-value = 0.001), with abnormal upper limb skeleton found highly significant in group D (p-value = 0.047) and group E (p-value = 0.009), and with abnormal lower limb skeleton found highly significant in group C (p-value = 0.009), group D (p-value = 0.028), and group E (p-value = 0.006). In contrast with the findings, it was evident that the greatest anomalies, particularly at higher doses of rolapitant, occurred in the experimental groups in comparison to control groups (Table 12).

**Table 12:** Lethal effect of different doses of rolapitant in experimental and control groups N = 30 in each group. a, b, c, d, and e: Control groups; A, B, C, D, and E: experiment groups. * indicates highly significant values.

Groups	Chick less than normal weight	Chick less than normal CRL	Skeletal abnormality in skull bones	Skeletal abnormality in vertebral column	Abnormal ribs	Abnormal sternum	Skeletal abnormalities in the bones of upper limb	Skeletal abnormalities in the bones of lower limb
a	1	1	0	0	0	0	1	1
A (p-value)	3 (0.3894)	2 (0.6404)	1 (0.5982)	1 (0.5982)	2 (0.1503)	1 (0.5982)	3 (0.3894)	3 (0.3894)
b	1	2	0	0	0	0	1	2
B (p-value)	3 (0.3894)	4 (0.4475)	1 (0.5982)	1 (0.5982)	3 (0.0756)	2 (0.1503)	2 (0.6404)	4 (0.4475)
c	2	3	2	1	2	2	2	2
C (p-value)	6 (0.3169)	8 (0.067)	3 (0.8919)	4 (0.1611)	5 (0.2276)	5 (0.2276)	6 (0.3169)	10* (0.009)
d	2	3	1	3	1	1	3	3
D (p-value)	10* (0.0283)	12* (0.0195)	6 (0.1317)	7 (0.1659)	8* (0.001)	7* (0.022)	9* (0.047)	10* (0.028)
e	3	4	1	2	2	1	3	5
E (p-value)	15* (0.0007)	16* (0.0029)	9* (0.0056)	8* (0.0377)	12* (0.002)	11* (0.001)	11* (0.009)	15* (0.006)

## Discussion

When a teratogen has an effect on the tissues of developing chick embryos, it causes morphological or functional abnormalities. This is referred to as a "teratogenic mechanism." A teratogen or one of its metabolites may induce the initial event in teratogenesis, with the teratogen affecting (a) an organ primordium that would subsequently be malformed, (b) embryonic tissues other than the malformed organ primordium, or (c) maternal tissue or placenta [13]. This study aimed to see how rolapitant affected the growth of developing chick embryos.

Across all doses in the current investigation, the mean weight and CR length of embryos were substantially greater in the control group than in the experimental group. The experimental group experienced a higher rate of death than the control group. Additionally, it was found that rolapitant's lethality increases with dose. When skeletal anomalies in the vertebral column are analyzed due to rolapitant's effects, all groups reveal abnormalities except group A. However, it was noted that it was more widespread in the experimental group and became more prevalent with repeated rolapitant dosages. Thus, the experimental group had the most anomalies, particularly at higher rolapitant doses. As shown in a review of teratological studies, “several factors contribute to abnormal cellular or subcellular development including chromosomal aberration, genetic mutation, mitotic interference, altered nucleic acid integrity or function, a lack of precursors and substrates, altered energy sources, enzyme inhibition, fluid osmotic imbalance, and altered membrane properties” [14].

Morphogenesis and tissue interaction are thought to be the fundamental regulators of differentiation and morphogenesis because they can occur between a pair of tissues or more with dissimilar attributes (heterotypic) or among the cells with similar properties (homotypic). Several cytotoxic drugs reduce cell quantity and impede cell proliferation, which is a key factor in the development of most deformities and teratogenesis [15]. Inhibition of RNA production and depletion of mucopolysaccharides, hydroxyproline, and phospholipids in the palatal shelves are the successive steps of this process [16].

The antiproliferative properties of cyclophosphamide, hypervitaminosis, amniocentesis, and chlorpromazine have all been investigated. Physical or chemical stress induces necrosis in tissues that are susceptible to anomalies in a couple of hours or days, according to numerous teratological studies. Higher doses result in cell death and teratogenesis, but lesser levels damage cells more slowly and may not cause any problems [17-20].

Cytotoxic substances affect cells differently. This could be due to several factors. There are several fundamental distinctions, the most important of which is the intrinsic cell distinction, which determines if a cell will live or die. A significant amount of vacuolation was detected in cells treated with 6-aminonicotinamides in ectodermal cells, although not in mesodermal or endodermal derivatives [21]. Diffusion indicates the nutritional condition of cells, which determines their sensitivity. The cells that are the farthest away from a nutrient supply are the ones that suffer the most [22].

The prescribing information for rolapitant hydrochloride (tablet Varubi) has been authorized by the US FDA, and it contains instructions on how to take the drug safely and effectively. Varubi's safety in emetogenic chemotherapy patients was investigated in four randomized clinical trials, including over 2800 patients. Varubi patients had greater adverse events in cycle 1 than the placebo patients. Major congenital impairments and miscarriages in clinically diagnosed pregnancies in the overall population of the United States were estimated. In the pregnant rats given during organogenesis, the potential toxicity of rolapitant hydrochloride to embryos and fetuses was studied. Rats treated with rolapitant showed signs of maternal toxicity in the first week, including a decrease in body weight gain and dietary intake. There were no teratogenic or embryo-fetal damaging findings in rabbits. During organogenesis and lactation, rats were given oral doses of 2.25, 9, or 22.5 mg/kg per day to see how rolapitant affected their development. When given at a daily dose of 22.5 mg/kg, the following side effects were observed: mortality/moribund state, decreased weight and dietary intake, entire litter loss, postponed parturition, shortened gestation length, and an increased number of unaccountable implantation sites (on a body surface area basis, approximately 1.3 times the approved intravenous human dose). Lower postnatal survival, lower body weights, and weight gain were all observed in the offspring, which could be linked to the reported maternal toxicity. In female pups, a maternal daily of 9 mg/kg rolapitant (about 0.5 times the maximum authorized intravenous human dose on a body surface area basis) resulted in memory loss and weight loss. There were fewer, mean implantation sites, corpora lutea, and viable embryos at 22.5 and 45 mg/kg/day (about 1.3 and 2.6 times the recommended intravenous human dose on a body surface area basis, respectively) compared to control [23].

Similarly, rolapitant decreased apomorphine-provoked retching as well as vomiting in ferrets in a dose-dependent manner, according to Duffy et al. [24]. Rolapitant suppressed acute and delayed period retching plus vomiting caused by the highly emetogenic chemotherapy agent cisplatin in ferrets at the same dose range. The NK1 antagonist aprepitant, on the other hand, is approved in humans for the management of CINV and postoperative nausea with vomiting (PONV) and is administered orally before and after chemotherapy [25]. Rolapitant appears to be a highly selective and competitive NK1 antagonist with good oral and CNS penetration, according to these results. Following their activation in humans, a number of NK1 antagonists have been found to have antiemetic effects [26].

Rojas et al. (2015) evaluated the mechanisms of action and the most recent clinical studies for rolapitant and netupitant/palonosetron (NEPA), two novel NK1 receptor antagonists used to treat chemotherapy-related nausea as well as vomiting. Throughout the delayed phase of all studies, patients who were given rolapitant had a considerably higher rate of full response than the patients who were given placebo. In addition, the study mentioned above looked into rolapitant's safety and tolerability. The most common TRAEs were fatigue, constipation, headache, diarrhea, dyspepsia, hiccups, and neutropenia (treatment-related adverse effects) [27]. Rapoport et al. (2016) studied rolapitant's efficacy and safety in avoiding nausea and vomiting caused by repeated rounds of weakly or highly emetogenic chemotherapy [28]. Rolapitant was shown to be widely tolerated above multiple MEC or HEC cycles, with minimal risk of treatment-emergent adverse effects (TEAEs) such as fatigue, constipation, headache, and weight loss. Schwartzberg et al. (2015) investigated the usage of rolapitant in cancer patients who had received moderately emetogenic chemotherapy or anthracycline and cyclophosphamide schedules. When used in conjunction with a 5-HT3 receptor antagonist and dexamethasone, rolapitant has been found to be well tolerated and to outperform active control in terms of reducing CINV for the entire five-day (0-120 h) at-risk period after the moderately emetogenic treatment followed by anthracycline and cyclophosphamide schedules after mildly emetogenic chemotherapy [29].

Small samples and the single-centric study was the limitation of the study. To our knowledge, no data on the rolapitant's effects on pregnant women were available. It is recommended that an extended study and a more detailed and thorough analysis should be done to strengthen the reliability and generalizability of the current investigation's findings. This research may aid in understanding the teratogenic effects of rolapitant in growing chick embryos. The study outcomes could help medics improve CINV by developing novel treatment alternatives.

Limitations of the study

In this work, we may have found information about teratogenic effects that are associated with massive doses of rolapitant administered to embryonic chicken, and these effects may be extended to humans. We have no data on the effects of rolapitant on pregnant women. Clinicians may find this study's findings beneficial in their quest to discover more effective ways to treat nausea and vomiting associated with chemotherapy. However, despite these flaws, the study has a demerit due to its small sample size and one-directional design. A large group study to enhance the constancy and generalizability of the current study’s observations is remanded by the author.

## Conclusions

The current study established that the effects of rolapitant increased the mortality rate, growth retardation, poor ossification, morphological deformity, and altered spinal column curvature. When excessive amounts of rolapitant are used, the substance poses hazards and deleterious effects. As a result, rolapitant should be used with caution only when a strong therapeutic indication criterion has been satisfied. Moreover, the drug has to be carefully monitored with all the clinical parameters and investigational profiles as recommended by the cancer protocols. It also reiterated that drug usage in pregnant women should be after necessary studies and approvals from regulatory agencies.
